# Management strategies of pediatric coccydynia: a narrative review

**DOI:** 10.3389/fped.2026.1750781

**Published:** 2026-03-09

**Authors:** Jinchao Cao, Kaixuan Tian

**Affiliations:** Department of Pediatric Orthopaedics, Hebei Medical University Third Hospital, Shijiazhuang, Hebei, China

**Keywords:** children, coccydynia, conservative treatment, etiology, pain management, surgical procedures

## Abstract

Pediatric coccydynia is a challenging, uncommon clinical entity that warrants management distinct from adult protocols, primarily due to the anatomical specificity of the developing coccyx. Despite its morbidity, current therapeutic decision-making is severely constrained by a critical lack of high-level evidence in the pediatric and adolescent population. This narrative review aims to address this knowledge gap by synthesizing the latest available literature on pediatric coccydynia to establish a contemporary, evidence-informed foundation for clinical practice. We performed a comprehensive search of in PubMed, Embase spanning from inception to the present, focusing on studies discussing the etiology, diagnosis, and management outcomes specifically in children and adolescents. The review structurally summarizes the distinguishing anatomy, diverse etiologies, and refined diagnostic approach relevant to pediatric coccydynia. The review outlines a potential stepwise treatment progression, beginning with non-operative strategies (including ergonomic adjustments, pharmacotherapy, and targeted nerve blocks) to coccygectomy as a reported surgical solution for refractory cases. This comprehensive synthesis offers a vital, evidence-informed framework for clinicians navigating the management of coccydynia in pediatric patients.

## 1 Introduction

Coccydynia, also known as coccygodynia or simply tailbone pain, is a debilitating pain syndrome characterized by discomfort or tenderness localized to the coccyx, the final segment of the spine (1). First termed coccydynia by Simpson in 1,859, and frequently referred to as the “lowest” site of low back pain, its underlying pathophysiology and accurate prevalence are still not fully understood. Although relatively rare in the general population and generally reported to be more prevalent in adults, particularly women, it can cause significant localized pain, often exacerbated by sitting, leading to a decreased quality of life (2). The coccyx's vulnerable location at the base of the spine makes it susceptible to various etiologies, ranging from common musculoskeletal injuries to rare causes like infections and malignancies.

While its precise incidence remains poorly documented in the literature, its substantial impact is evidenced by over 14,000 annual emergency department visits and more than 1,300 coccygectomies performed each year in the USA (2). The condition disproportionately affects adults, with a reported mean onset age of 40 years and a five-fold higher prevalence in women compared to men (3). In sharp contrast, coccydynia is considered relatively rare in the pediatric population, accounting for less than 1% of all childhood low back pain cases (4). This low incidence, coupled with the fundamental differences in etiology, pathophysiology, and treatment requirements between adult and pediatric presentations, underscores the necessity of a dedicated review focusing on tailbone pain in children.

Coccydynia in the pediatric patient presents a unique clinical and anatomical challenge distinct from the adult form. Fundamentally, the specificity of pediatric coccydynia lies in its immature skeletal structure; unlike the often-fused adult coccyx, the pediatric coccygeal region is composed of three to five unfused vertebral segments, making it inherently more vulnerable to external mechanical trauma or chronic strain (5). This developmental state, coupled with a dense neurovascular distribution, means that nerve root compression or localized inflammatory responses are potentially heightened pain generators (6). While many pediatric cases are self-limiting, others are notoriously persistent and difficult to treat, often complicated by the lack of a specific diagnosis. Thus, effective clinical management may benefit from a specialized approach that thoroughly accounts for these distinctive anatomical and physiological features of the growing spine. In this review, priority was given to literature specifically involving pediatric and adolescent subjects. Methods or treatments with no reported clinical application in children were excluded, and findings primarily derived from adult cohorts are clearly distinguished to prevent over-generalization.

The management of pediatric coccydynia currently operates within a significant knowledge gap, as the vast majority of existing clinical cohort studies and treatment recommendations are based solely on the adult population (7). Consequently, therapeutic strategies for children often rely on extrapolations from adult-focused protocols, despite the recognition that pediatric coccydynia is characterized by complex and diverse etiologies, including trauma, infection, and, notably, a higher predisposition to congenital anomalies (such as sacrococcygeal teratomas) and developmental abnormalities (like spina bifida) compared to adults. The diagnostic and treatment strategies for children must therefore be highly individualized, factoring in the patient's age, specific etiology, and pain severity. This narrative review aims to address these deficiencies by synthesizing the available knowledge on etiology, pathogenesis, and therapeutic progress, providing a comprehensive reference for clinicians and establishing future research directions to advance the field beyond adult-centric treatment paradigms.

Given the rarity of coccydynia in children, this narrative review adopted a comprehensive search strategy across PubMed, EMBASE, Web of Science, and Google Scholar for literature published through October 2025. Keywords prioritized ‘pediatric coccydynia,’ ‘childhood tailbone pain,’ and associated anatomical and management terms. Due to the paucity of prospective pediatric trials, we intentionally utilized selective citation of significant case series and adult-derived protocols with established clinical relevance to children. This methodology facilitates a robust synthesis of the current therapeutic landscape while maintaining transparency regarding the limited volume of primary pediatric data.

## 2 Anatomy of coccyx

The term coccyx originates from the Greek word for “cuckoo bird's beak,” a nod to the bone's distinctive lateral profile. Although commonly known as the tailbone, the structure is highly variable and typically consists of three to five distinct coccygeal vertebrae, with four segments present in the majority of subjects (70%–80%) (8). The superior segment, Co1, is the most substantial, featuring vestigial articular processes (coccygeal cornua) that delineate the inferior boundary of the sacral hiatus. Co1 articulates with the sacrum primarily through the sacrococcygeal joint, a symphysial joint stabilized by fibrocartilage and often supplemented by bilateral zygapophysial (facet) joints. Furthermore, the transverse process of Co1 may interface with the transverse process of S5, effectively forming the fifth anterior sacral foramen which houses the anterior division of the S5 nerve. These sacrococcygeal and intra-coccygeal articulations are often considered functionally significant, allowing for a modest degree of motion—specifically forward flexion—during weight-bearing actions such as sitting. However, the degree of joint integrity is highly inconsistent; clinical studies have indicated that the sacrococcygeal joint is fused in a substantial proportion of the population, reported to be around 63% (8).

The coccyx functions as a primary anchor for numerous muscular and ligamentous structures, contributing to pelvic floor stability and function. Muscular attachments are distributed across its surfaces: the anterior aspect provides insertion points for key pelvic floor components, including the levator ani, iliococcygeus, coccygeus, and pubococcygeus. Conversely, the posterior surface provides a significant origin or insertion site for the large gluteus maximus muscle (9). Stabilization of the sacrococcygeal joint is achieved through a complex network of ligaments. The Lateral Sacrococcygeal Ligament connects the transverse process of the first coccygeal vertebra (Co1) to the inferolateral border of the sacrum, an attachment critical for completing the foramen that transmits the fifth sacral nerve (S5). The Posterior Sacrococcygeal Ligament consists of deep and superficial components; the deep portion continues the posterior longitudinal ligament inside the sacral canal, while the superficial portion descends from the sacral hiatus. Additionally, an Intercornual Ligament spans between the sacral and coccygeal cornua. The neuroanatomy of the region is primarily associated with the coccygeal plexus, which is primarily derived from the ventral rami of the fourth (S4) and fifth (S5) sacral nerves along with the coccygeal nerve (Co) (7). Despite the clinical prevalence of coccydynia, the precise contribution of these terminal coccygeal nerves to the pathology is often overlooked in current literature.

The fundamental anatomical principle that “children are not little adults” is critical for clinicians evaluating pediatric coccydynia, requiring a distinct understanding of coccygeal development. The coccyx matures through a relatively simple, yet highly variable, developmental process involving the ossification of up to eight centers, resulting in the structure's final, inconsistent segmentation (10). Radiologically, the postnatal morphological development of the coccyx in children aged 1–18 years can be divided into three phases (11): Stage 1 (Infancy to Early Childhood) is characterized by the structure's shortest length, remaining cartilaginous until approximately age two, with ossification initiating in the first segment (Co1) around age three. Stage 2 (Late Childhood to Prepubescence) sees the coccyx attain medium size; Co1 is fully ossified by age six, and ossification of all lower segments is completed by approximately age 11. The final phase, Stage 3 (Postpubescence), is when the coccyx reaches its full adult size. Anatomically, this process is segment-specific (9): Co1 forms from a centrum and two neural arches, while the remaining caudal vertebrae (Co2, Co3, Co4) are formed only by centra, which ossify in a craniocaudal direction between 16 months and 18 years of age. Unlike other skeletal regions, the fusion of these coccygeal segments is highly asynchronous, often beginning around age six and potentially continuing into the third decade of life (up to 30 years). Understanding this extensive variability in the timing of ossification and fusion is helpful to avoid the misinterpretation of normal developmental anatomy as pathology or instability.

## 3 Etiology

The etiology of coccydynia in children and adolescents is multifaceted and aligns with the biopsychosocial model of pediatric pain. While biological factors remain prominent—with trauma (e.g., backward falls, cycling, or dancing) being the primary cause—pain is also frequently linked to abnormal morphology, such as excessive angulation or distal bone spurs. Abnormal Mobility, detected through dynamic radiography, is considered a significant factor, manifesting as either hypermobility (excessive movement, often >25° upon sitting) or hypomobility (a rigid coccyx, <5° movement). Other causes, which should be clinically considered and excluded where appropriate, include serious conditions like infections (osteomyelitis, pilonidal cysts), inflammatory disorders, or, rarely, malignancies. However, up to 40% of cases are idiopathic, underscoring the necessity of considering psychological and social stressors (such as school-related stress or family dynamics) that may modulate pain perception and chronicity in this sensitive population.

### 3.1 Trauma

Trauma is consistently identified as the dominant etiology of coccydynia in both adults and adolescents (12). Functionally, the coccyx acts as the third leg of the supporting tripod, alongside the ischial tuberosities, making it inherently vulnerable to external injury, most commonly from a direct backward fall (13). Studies in the literature report a high incidence of antecedent direct trauma, found in 50%–65% of adult patients and noted in 62% of adolescents presenting with the condition (13, 14). Injuries can range in severity from a mild sprain of the pelvic floor muscles or simple coccygeal distortion to a severe fracture-dislocation of the sacrococcygeal complex.

In the pediatric and adolescent populations, the developmental anatomy is a key consideration; the coccyx matures through asynchronous ossification and fusion, which can complicate the interpretation of bony trauma and instability. Beyond acute incidents, chronic microtrauma is a regarded as predisposing factor, often resulting from prolonged sitting on hard or poorly cushioned surfaces. This mechanism, where poor postural adaptation leads to chronic pain, was historically recognized by Schapiro (15) in 1950, who referred to coccydynia as the “television disease.” This highlights the relevance of blunt, repetitive trauma in children and adolescents who spend extended periods seated.

### 3.2 Coccygeal morphology

**Figure 1:**
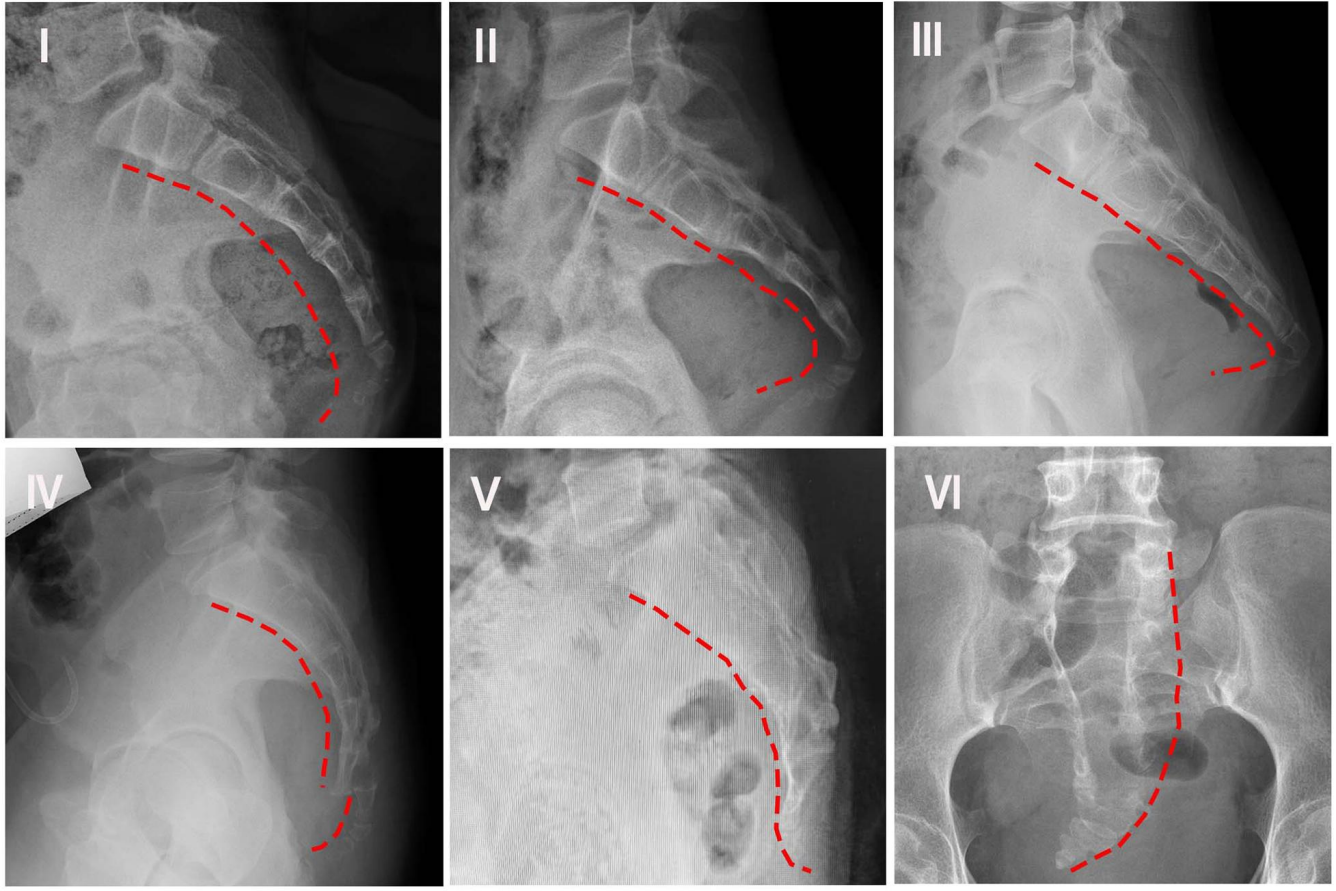
Morphology of coccyx types I–VI, as categorized in Table 1.

Variations in the anatomical shape of the coccyx are considered a potential cause of coccydynia, distinct from the theorized role of its mobility. The fundamental classification of coccygeal morphology was initially established by Postacchini and Massobrio in 1983, which defined four primary configurations (Types I–IV) (16). This system was subsequently expanded by Nathan et al. in 2010 to include two additional variants (Types V and VI), resulting in the six-type classification currently used in clinical practice (Table 1; Figure 1) (17).

**Table 1:** Morphology of the coccyx by Nathan (17).

Type	Coccygeal morphology
Type I	Gentle ventral curvature with the apex pointing caudally
Type II	Marked curve with the apex pointing straight forward
Type III	Acute anterior angulation without subluxation
Type IV	Anteriorly subluxated at the level of the sacrococcygeal joint or first or second intercoccygeal joint
Type V	Coccygeal retroversion with spicule
Type VI	Scoliotic or laterally subluxated coccyx

Clinical evidence consistently links specific morphological types to increased pain risk. Type I morphology is characterized by a gentle ventral curvature with a caudally pointed apex and is considered the normal variant, found in over 50% of the population (8). However, subsequent studies demonstrate a shift toward less common variants in patient cohorts. In a case-control study (*n* = 107), Woon et al. (18) observed that while Type I and Type II were the most frequent variants in affected individuals (e.g., Type I in 34% of females and 50% of males), the overall patient group showed a lower prevalence of Type I but a significantly higher proportion of Type II morphology compared to pain-free controls. This association was strongly reinforced by Shams et al. (8) in a case-control study of 60 patients with idiopathic coccydynia, which reported a Type II coccyx in 50 patients (83.3%)—a rate substantially higher than the 40% observed in the control group without the condition.

Furthermore, a prospective clinicoradiological observational study (*n* = 168; 106 females, 62 males) indicated that Type II and Type IV were the most common coccyx morphologies encountered in patients with chronic coccydynia (19). This study also provided prognostic insights, noting that outcomes were better in patients with a traumatic etiology compared to those with an idiopathic etiology, and in patients presenting with pseudoarthrosis visible on MRI.

### 3.3 Coccyx mobility

In addition to variations in coccygeal morphology, abnormal mobility of the coccyx has been proposed as another potential etiology of coccydynia. The coccyx, situated at the most distal segment of the vertebral column, forms a tripod-like support structure together with the ischial tuberosities. It serves as an attachment site for multiple muscles and ligaments that contribute to weight distribution during sitting and provide mechanical support to the pelvic floor (20). Coccygeal instability is characterized by excessive movement between one or more coccygeal segments. This instability is often “dynamic,” meaning it becomes apparent only under specific mechanical loads, such as during maximal weight-bearing in a seated or partially reclined position—a common aggravating posture in individuals with coccydynia (21). Several studies support the concept of coccydynia as a dynamic rather than static condition (22, 23). For instance, abnormal translational or angular motion of the coccyx has been observed in up to 69% of patients with coccydynia using dynamic radiographs taken in both standing and seated positions (24).

Maigne et al. (24) developed a classification system for coccygeal mobility, categorizing it into four types: luxation (anterior or posterior dislocation), hypermobility, immobility, and normal mobility (see Table 2). In a follow-up study of 208 patients with coccydynia, the authors reported posterior luxation in 22.1% of cases, anterior luxation in 5.3%, hypermobility in 27.4%, and a bony spicule in 14.4%. Only 30.8% of the patients exhibited normal coccygeal mobility.A study focusing on 53 adolescents with chronic coccydynia revealed a different pattern: abnormal mobility was less common compared to adults, while bony spicules were more frequent (*P* < 0.001). Among these adolescents, 11 out of 27 MRI scans showed a hypersignal within the disc or adjacent bone, and 6 out of 27 exhibited a hypersignal around the tip of the coccyx, suggestive of bursitis (4).

**Table 2:** Maigne classification of coccygeal mobility.

Type	Dynamic radiography criteria	Clinical associations & characteristics
Type Ⅰ: Luxation	Posterior subluxation of the mobile coccygeal segment upon assuming a sitting posture.	More commonly associated with obese individuals, resulting from increased intra-pelvic pressure
Type Ⅱ:Hypermobility	Coccygeal flexion greater than 25° when sitting.	More common cause of pain in thin individuals; may be isolated or associated with a bone spicule
Type Ⅲ:Immobile	Coccygeal flexion less than 5° when sitting	May cause coccydynia either in isolation or in combination with a posterior spicule
Type Ⅳ:Normal mobility	Coccygeal mobility between 5° and 25° on dynamic radiography	Normal physiological range, generally not associated with pain

### 3.4 Others

Beyond the common traumatic or postural causes, the literature documents a number of rarer etiologies contributing to coccydynia, encompassing structural, metabolic, infectious, and neoplastic factors (12, 25, 26). Obesity is a well-established predisposing factor; increased body mass is biomechanically linked to restricted sagittal pelvic rotation while sitting, leading to the protrusion and retroversion of the coccyx tip, thus subjecting it to excessive pressure. Studies indicate a significant risk increase in females with a BMI over 27.4 and males over 29.4 (12). Furthermore, in cases where the physical examination lacks the characteristic local tenderness, the pain may be radicular or referred in nature, often originating from upper lumbar disc herniation, lumbosacral nerve root compression, or Pudendal Neuralgia caused by chronic microtrauma (e.g., cycling). Less commonly, acute pain can be attributed to calcium salt deposition (e.g., apatite or CPPD) in the sacrococcygeal joint or surrounding tendons (26).

Infectious and neoplastic processes, though rare, necessitate careful consideration. Infection should be strongly suspected if symptom onset coincides with recent local surgical intervention, or if a Pilonidal Cyst/Sinus becomes infected, manifesting as severe pain and localized swelling (26). Though uncommon, neoplasms are critical differential diagnoses; tumors like chordomas or chondrosarcomas can directly invade the region (27). Additionally, benign cysts can cause significant pain, notably Tarlov Cysts, which are cerebrospinal fluid-filled perineurial cysts most frequently affecting the sacral nerve roots (S2 level) (28). Expansion of these cysts leads to compression of the contained or adjacent nerve roots, triggering symptoms. Finally, after the exclusion of organic pathology, pain may be associated with nonorganic causes, such as somatization or other psychological disorders (17).

## 4 Diagnosis

Coccydynia is characterized by localized pain and intense tenderness in the coccyx and surrounding soft tissues. While symptoms can sometimes be confused with referred pain from the lumbar spine, a thorough clinical and physical examination is widely regarded as a fundamental component of the diagnostic process (29). Key findings include severe tenderness upon direct palpation and during intrarectal manipulation of the coccyx, as well as possible hyper- or hypo-mobility. The physical examination consistently reveals immediate, exquisite tenderness upon direct palpation of the coccyx. A careful inspection of the overlying skin is also mandatory to exclude secondary causes such as pilonidal disease, other cutaneous lesions, or masses that could suggest an underlying infectious or neoplastic process.

Initial diagnostic imaging should begin with static and dynamic stress x-rays (standing and sitting lateral views) to rule out fractures, malignancy, or secondary conditions like pilonidal cysts, and to detect the frequent underlying cause of sacro-coccygeal joint hyper-mobility or subluxation (30). However, since static images frequently fail to capture the mechanical instability associated with coccydynia, one of the most insightful initial investigations frequently utilized involves dynamic stress x-rays, taken in both standing and sitting lateral positions (5). This technique is considered highly effective by many clinicians, demonstrating sacro-coccygeal joint hyper-mobility or displacement in a significant proportion of patients, a finding that is notably absent in asymptomatic individuals. Although static imaging (standard x-rays, CT, and MRI) is often inconclusive regarding pain etiology, it remains essential for excluding serious pathology (31). CT scans are recommended for definitive diagnosis of trauma-related fractures, while MRI offers superior visualization of morphology and soft tissues, helping identify inflammation (increased T2 signal) associated with chronic joint instability. Ultrasound is playing an increasingly pivotal role in clinical practice, and the sacrococcygeal region is no exception; a study of 805 children demonstrates that sacrococcygeal ultrasonography may reliably identify anatomical variations while eliminating radiation exposure (32). Ultimately, the diagnosis of coccydynia relies on correlating the patient's specific symptoms and findings from the physical examination with imaging results to exclude other conditions (33).

## 5 Nonoperative treatment

Managing coccydynia in children usually begins with a conservative strategy, favoring approaches that are non-surgical and minimally invasive. In alignment with the gold-standard biopsychosocial model, management is encouraged not only target biological symptoms but also address the psychological and social dimensions of the child's life. A common framework fortreatment involves comprehensive ergonomic adjustments—like using specific cushions and managing weight—alongside focused medication (chiefly NSAIDs) and specialized manual and physical therapies to ease muscle tension and improve function. Furthermore, psychological screening and support may be integrated to manage pain-related anxiety or school-based stressors that contribute to pain chronicity. If symptoms persist despite these efforts, clinicians may consider interventional pain techniques, such as image-guided steroid injections or nerve blocks, which deliver effective, localized relief. While most of the supporting clinical data comes from studies on adults, these conservative methods are widely accepted as the safe and effective starting point for treating coccydynia in adolescents and children. While conservative management is widely reported to be effective, it is important to note that most available pediatric evidence stems from small retrospective cohorts (Level IV evidence). The high success rates reportedshould be interpreted with caution due to the potential for recall bias and the lack of standardized, prospective control groups in these studies.

### 5.1 Ergonomic adaptation

The prognosis for many patients suffering from coccydynia is often favorable, with a significant proportion experiencing spontaneous symptom resolution within weeks or months of onset, irrespective of formal medical intervention. Indeed, conservative treatment approaches have been reported in various cohorts to achieve remarkable success rates, sometimes exceeding 90% (34). A primary strategy of management involves comprehensive ergonomic adaptation and lifestyle modification. This includes offering weight loss strategies to all affected obese patients, as reducing overall body mass can significantly lessen the load on the tailbone. Furthermore, patient education on adopting and maintaining optimal sitting posture is often beneficial, as correcting chronic postural faults can mitigate underlying contributing factors (26).

Recognizing the “social” aspect of the biopsychosocial model, clinicians should also provide guidance for school environments, such as recommending scheduled standing breaks during long classes or the use of portable cushions in classrooms to prevent social withdrawal due to pain (35). The use of specialized seating aids is frequently utilized in managing discomfort. While doughnut-shaped cushions are frequently recommended, they are often subpar for coccydynia as their design can inadvertently increase pressure on the coccyx. Consequently, a frequently suggested ergonomic solution involves U-shaped or wedge-shaped cushions which feature a strategic cut-out at the rear. This design facilitates the coccyx to effectively “hover” over the empty space, thereby eliminating direct contact and resulting in reduced coccygeal weight-bearing and pain relief (20, 36). Crucially, for pediatric coccydynia, this multi-faceted ergonomic adaptation—encompassing postural modification, weight management where applicable, and the correct selection of pressure-relieving cushions—constitutes the preferred initial line of treatment for alleviating clinical symptoms.

### 5.2 NSAIDs

Pharmacological intervention, primarily centered on Nonsteroidal Anti-inflammatory Drugs (NSAIDs), forms a key component of the preferred initial conservative management strategy for coccydynia. Both oral and topical NSAIDs are valuable in the acute phase, aiming to concurrently reduce pain and localized inflammation (26)**.** Clinical evidence supports this approach; a retrospective review by Hodges et al., for instance, indicated that 66% of patients reported pain improvement through NSAID monotherapy (37). Furthermore, long-term studies of adolescent cohorts reinforce this positive trend: a series of 53 adolescent patients treated conservatively showed favorable outcomes over a four-year follow-up (4). While systemic medications such as oral NSAIDs (e.g., ibuprofen), acetaminophen, and opioid analgesics (e.g., oxycodone and hydrocodone) can offer generalized relief, their primary limitation is their systemic distribution, which results in minimal focal activity directly at the coccyx. This systemic action also carries the risk of undesirable gastrointestinal and other systemic side effects. Consequently, while oral NSAIDs are useful, topical formulations may offer a more focused alternative to alleviate local coccydynia symptoms with potentially fewer systemic risks.

### 5.3 Manual therapy

Manual therapy, commonly performed by chiropractors or physical therapists, is a well-reported treatment modality for coccydynia, fundamentally comprising massage and manipulation (38–40). Specific manual techniques include levator ani massage, levator ani stretch, and joint mobilization, which can be performed externally or internally via the per-rectal route. This approach targets the pathogenesis of pain, where levator ani spasm plays a pivotal role, exacerbating sacro-coccygeal joint issues and creating a vicious pain cycle that massaging helps break (22). In terms of long-term outcomes, levator ani stretching and massage have been shown to yield sustained improvements in pain, often surpassing the efficacy of joint mobilization (1). However, despite its reported success, systematic reviews suggest that while manual therapy demonstrates efficacy for acute cases in adults, its effectiveness for chronic pain requires more robust pediatric-specific investigation (41).

For chronic symptoms, specialized techniques, such as the transrectal approach, offer significant symptom relief by relaxing tense pelvic floor muscles or mobilizing a stiff coccyx (42). This manipulation is vital for specific patient subsets, including those with abnormal coccygeal mobility but minimal spasm, where manipulation and post-manipulation extension are recommended (42). Recent clinical studies further support these methods: Osteopathic Manipulative Treatment (OMT) has demonstrated a successful and significant reduction in pain and disability in chronic coccydynia compared to conventional therapy (38). Consistent with this, transrectal OMT has been confirmed as a feasible and highly acceptable technique in primary care, resulting in significant immediate and follow-up self-reported pain improvement (43). All current clinical findings are based on adult cohorts, highlighting a need for further research specifically targeting children and adolescents.

### 5.4 Physical therapy

Physical therapy (PT) interventions, which encompass modalities such as Extracorporeal Shock Wave Therapy (ESWT) and kinesiotaping combined with therapeutic exercise, have demonstrated significant short-term efficacy in achieving pain reduction and improving functional status for patients with coccydynia. Kinesiotaping, utilized as an adjuvant therapy, specifically contributes to enhanced trunk mobility when paired with exercise. Furthermore, pelvic floor physical therapy (PFPT) is uniquely beneficial for patients whose symptoms are driven by substantial muscular pain originating from the adjacent para-coccygeal musculature, such as the levator ani. This targeted approach aims to alleviate the myofascial tension that contributes directly to coccygeal discomfort.

Among these modalities, ESWT has emerged as a particularly promising conservative treatment option, with evidence suggesting that tailoring shockwave therapy parameters to the patient's specific pain tolerance can enhance long-term success rates and mitigate the risk of recurrence. A systematic review further reinforces the potential of physiotherapy interventions, particularly ESWT, by concluding they are promising conservative treatments that significantly reduce pain and improve function in coccydynia. However, the systematic conclusion noted significant limitations, including high methodological variability, small sample sizes, and a generalized lack of long-term follow-up data (41). Despite these evident limitations, a retrospective cohort study involving patients aged 16 and older demonstrated strong clinical effectiveness: ESWT provided durable pain control for chronic coccydynia, resulting in a marked decrease in the mean Visual Analogue Scale (VAS) score (from 9.6 to 3.4) and overall improved quality of life at a 6-month follow-up (44). Crucially, the current body of evidence is exclusively based on adult or near-adult populations, highlighting a significant research gap concerning the application of these physical therapy modalities in pediatric and adolescent cohorts.

### 5.5 Injections and nerve block

Targeted interventional pain management is a pivotal strategy for treating pediatric refractory coccydynia, relying on techniques like fluoroscopy-guided local corticosteroid injections and sympathetic nerve blocks to deliver high concentrations of therapeutic agents precisely to the underlying pain generators, thereby minimizing systemic side effects. Crucially, for children undergoing these invasive needle procedures, the consideration of sedation is essential. Procedural sedation not only mitigates pain-related anxiety and distress but also ensures the child remains immobile, thereby enhancing the safety and precision of the intervention. Corticosteroid injections, ideally suited for acute or subacute cases (symptoms under six months), are precisely placed under imaging guidance at sites of instability, such as the sacrococcygeal joint or distal bone spurs, while the Ganglion Impar block specifically addresses the neuropathic and sympathetic pain component originating from the pelvic floor (4, 45). Although fluoroscopy is the standard for maximizing accuracy, some clinicians, based on established protocols, utilize digital intrarectal control to guide injections to the most symptomatic level (e.g., Co1-Co2) (46). Clinical data supports the efficacy of these procedures; for instance, in one series, a total of 27 patients received injections, and results from a study of 28 treated adolescents indicated a 50% success rate (seven completely well, seven much better), demonstrating that the symptomatic improvements achieved are comparable between juvenile and adult populations (47). However, significant heterogeneity exists in the literature regarding the types of corticosteroids used, the precise anatomical approach, and the criteria for “success.” This variability makes it challenging to establish a universal protocol for pediatric nerve blocks.

The ganglion impar, a critical sympathetic nerve target located adjacent to the sacrococcygeal joint, is a highly effective target for interventional pain management, consistently demonstrating a relatively higher reported clinical efficacy compared to other techniques for refractory coccydynia and chronic perineal pain. Ganglion impar block(GIB) is invaluable for both evaluating and managing pain maintained by sympathetic involvement in the coccyx and perineum. Clinical evidence strongly supports its efficacy, especially when utilizing Radiofrequency (RF) techniques. Studies employing Pulsed Radiofrequency (PRF) and Conventional Radiofrequency (CRF) ablation of the GI report high success rates, often exceeding 80% for chronic pain conditions, with outcomes proving particularly superior in patients presenting with a neuropathic pain component (48–50). To ensure safety and optimal placement, the procedure is typically performed with the assistance of imaging guidance (like fluoroscopy) using various needle approaches (29). The GIB and its radiofrequency variants are established as versatile and effective interventional treatments for chronic coccydynia and perineal pain, demonstrating success across a wide range of patients, from adolescents to the elderly (46, 51). A case report documented the successful treatment of chronic, disabling idiopathic coccydynia in a 15-year-old adolescent using a GIB after conservative therapies had failed, resulting in significant and persistent pain improvement (52). Studies spanning ages 15–75 affirm that Ganglion Impar Pulsed Radiofrequency (GI-PRF) is a safe method, with 75% of chronic pain patients achieving at least a 50% reduction in their Visual Analogue Scale (VAS) scores (50). Furthermore, Conventional Radiofrequency (CRF) ablation has shown superior results in adolescents with chronic non-oncological perineal pain, achieving excellent outcomes and significant pain reduction in 82% of patients after six weeks (53). The utility of GIB even extends to pediatric oncology, as documented by a successful phenol neurolysis on a 3-year-old patient with refractory perineal cancer pain, illustrating its role in managing sacrococcygeal pain across the life span (54). In such young or medically complex pediatric cases, a tailored sedation or anesthetic protocol is fundamental to the successful execution of the block. Given the severity of reported rare complications, such as a documented case of conus infarction following a non-guided block, clinicians must prioritize safety by strictly adhering to the recommendation of using imaging guidance to minimize the risk of inadvertent intravascular injection (55). Targeted interventional pain management, primarily involving fluoroscopy-guided corticosteroid injections and GIBs is an effective and safe strategy for refractory coccydynia across all ages, including pediatric and adolescent patients, provided strict imaging guidance is used to mitigate rare but serious neurological risks. First documented by Klocke et al. in 2003 (56), ultrasound-guided caudal block has become increasingly prevalent in clinical practice. Its efficacy is well-supported by studies across various ethnicities, which report near-perfect success rates (57, 58). In light of the risks associated with radiation exposure, ultrasound guidance emerges as a particularly advantageous modality for pediatric patients.

## 6 Coccygectomy

Despite the implementation of these conservative strategies, persistent coccydynia may necessitate surgical consideration. In these difficult-to-treat cases, the most common surgical option is coccygectomy, which involves partial or total removal of the coccyx. This treatment is generally reserved for the small percentage of patients who fail to achieve adequate relief from non-surgical care. Partial or total coccygectomy has been described as a potential intervention in both traumatic and idiopathic coccydynia after conservative measures have not achieved desired outcomes. However, postoperative complications following coccygectomy can include local infection, pelvic floor prolapse (sagging), retained coccygeal fragments, and persistent pain despite the surgery. To address these concerns and minimize complications, some authors have proposed novel surgical approaches. Specifically, one technical note on ten patients found that the Z-plasty technique for wound closure may help prevent wound dehiscence and surgical site infections, yielding favorable outcomes in that specific group (59).

Retrospective data suggests a favorable success rate for coccygectomy, generally ranging from 70% to 71%, with reported patient satisfaction rates as high as 89% at follow-up. This procedure has been shown to offer superior and sustained improvements in pain, functional status, and quality of life compared to continued conservative treatment (60, 61). Furthermore, comparative analysis suggests that patients with a traumatic etiology may achieve more favorable functional outcomes following surgery than those with an atraumatic cause (62). While the procedure is effective, certain preoperative factors, such as psychiatric illness, higher baseline pain scores, poor quality of life, and opiate use, have been identified as predictors of treatment failure (63). Additionally, technical modifications, such as utilizing a paramedian surgical approach instead of the traditional midline incision, have been demonstrated to maintain efficacy while significantly lowering the risk of postoperative complications (64).

The reported efficacy of coccygectomy extends to the pediatric and adolescent population, providing a viable option for those with chronic coccydynia refractory to conservative management. Multiple studies support this potential intervention: a case series involving eight pediatric and adolescent patients (aged 7–15) demonstrated favorable results, with excellent or good outcomes reported in all cases following total or partial coccygectomy (65). Retrospective analysis, including cohorts of adolescents, indicates an overall success rate ranging from 68% to 76% for the procedure in patients unresponsive to non-operative treatment (47, 66). Specifically, one large prospective cohort study, which included adolescents as young as 15, showed significant postoperative improvements in self-reported pain, mobility, and quality of life at one year, achieving a 70% overall patient satisfaction rate (67).

To mitigate the inherent morbidity risks associated with traditional open coccygectomy, novel, minimally invasive surgical techniques have been recently introduced, including endoscopic approaches and coccygeoplasty. Endoscopic coccygectomy was first reported in 2022 as a minimally invasive option for chronic refractory coccydynia. For instance, Roa et al. described the use of a minimally invasive endoscopic approach to perform complete coccygectomy in adult patients (68). However, its application in pediatric and adolescent patients remains to be established as it has not yet been reported in this age group.Beyond resection, other surgical management options have emerged. This includes the percutaneous injection of polymethylmethacrylate bone cement into acutely fractured coccyges, a procedure termed “coccygeoplasty,” as reported by Dean et al. and Akar et al., with patients achieving post-operative pain relief and improved functional outcomes (69, 70). Coccygeoplasty has garnered recent attention as a minimally invasive technique, particularly for coccydynia linked to coccygeal hypermobility. One study evaluating this procedure found it to be safe and effective, with the majority of patients (75%) reporting significant and sustained pain reduction at both 3- and 12-month follow-ups, independent of the final radiographic appearance (71). Nevertheless, due to the complete absence of published data in the pediatric or adolescent population, this procedure is not currently recommended for use in younger patients.

## 7 Strengths and limitations

This narrative review provides a much-needed synthesis of management for pediatric coccydynia; however, several limitations merit consideration. The primary limitation is the scarcity of high-level, pediatric-specific evidence (Level I or II). Most recommendations are based on retrospective case series (Level IV) or extrapolated from clinical trials. Additionally, the small sample sizes in available pediatric cohorts and the lack of standardized outcome measures across studies introduce significant heterogeneity. These factors limit our ability to perform a meta-analysis or provide definitive recommendations. Future research should focus on prospective, multi-center registries to better define the natural history and optimal treatment thresholds in the pediatric population.

## 8 Conclusion

Managing pediatric coccydynia requires a specialized, stepwise strategy with increasing invasiveness that fundamentally differs from adult protocols due to the anatomical nuances of the developing coccyx. The immediate priority lies with conservative care, employing ergonomic adaptation, targeted pharmacotherapy, and physical therapy. This narrative review highlights the progressive nature of treatment for pediatric coccydynia, emphasizing non-invasive and minimally invasive measures as the primary therapeutic mainstays. Current therapeutic knowledge remains heavily extrapolated from adult data, therefore, future research must prioritize large-scale randomized controlled trials within the pediatric population to establish personalized, evidence-based treatment guidelines.

## References

[1] Sidiq M Ravichandran H Janakiraman B Chahal A Rai RH Alotaibi AH Effectiveness of physical therapy interventions for coccydynia: a systematic review with a narrative synthesis. Arch Physiother. (2025) 15:77–89. 10.33393/aop.2025.3233 40308532 PMC12042952

[2] Woon JTK Stringer MD. Clinical anatomy of the coccyx: a systematic review. Clin Anat. (2012) 25(2):158–67. 10.1002/ca.21216 21739475

[3] White WD Avery M Jonely H Mansfield JT Sayal PK Desai MJ. The interdisciplinary management of coccydynia: a narrative review. PM R. (2021) 14(9):1143–54. 10.1002/pmrj.12683 34333873

[4] Maigne JY Pigeau I Aguer N Doursounian L Chatellier G. Chronic coccydynia in adolescents. A series of 53 patients. Eur J Phys Rehabil Med. (2011) 47(2):245–51. 21597433

[5] Skalski MR Matcuk GR Patel DB Tomasian A White EA Gross JS. Imaging coccygeal trauma and coccydynia. Radiographics. (2020) 40(4):1090–1106. 10.1148/rg.2020190132 32609598

[6] Sukun A Cankurtaran T Agildere M Weber M-A. Imaging findings and treatment in coccydynia - update of the recent study findings. Rofo. (2023) 196(6):560–72. 10.1055/a-2185-8585 37944937

[7] Daily D Bridges J Mo WB Mo AZ Massey PA Zhang AS. Coccydynia: a review of anatomy, causes, diagnosis, and treatment. JBJS Rev. (2024) 12(5):e24. e24.00007 10.2106/JBJS.RVW.24.00007 38709859

[8] Shams A Gamal O Mesregah MK. Sacrococcygeal morphologic and morphometric risk factors for idiopathic coccydynia: a magnetic resonance imaging study. Global Spine J. (2023) 13(1):140–8. 10.1177/2192568221993791 33567908 PMC9837515

[9] Broome DR Hayman LA Herrick RC Braverman RM Glass RB Fahr LM. Postnatal maturation of the sacrum and coccyx: mR imaging, helical CT, and conventional radiography. AJR Am J Roentgenol. (1998) 170(4):1061–1066. 10.2214/ajr.170.4.9530059 9530059

[10] Standring S. Gray’s Anatomy: the Anatomical Basis of Clinical Practice. 42nd ed. London: Elsevier (2020).

[11] Meylani N Ten B Temel G Yüksek HH Cömert AD Beger B Morphologic evaluation of the coccyx in the pediatric population. Surg Radiol Anat. (2025) 47(1):147. 10.1007/s00276-025-03662-4 40419789

[12] Maigne JY Doursounian L Chatellier G. Causes and mechanisms of common coccydynia: role of body mass index and coccygeal trauma. Spine (Phila Pa 1976). (2000) 25(23):3072–79. 10.1097/00007632-200012010-00015 11145819

[13] Kodumuri P Raghuvanshi S Bommireddy R Klezl Z. Coccydynia - could age, trauma and body mass index be independent prognostic factors for outcomes of intervention? Ann R Coll Surg Engl. (2017) 100(1):12–5. 10.1308/rcsann.2017.0089 29260897 PMC5838659

[14] Allahverdi E Allahverdi TD. A retrospective study on the pathologies in patients with coccydynia-lumbosacralgia and their treatment. Asian J Surg. (2022) 46(2):688–91. 10.1016/j.asjsur.2022.06.078 35843825

[15] Schapiro S. Low back and rectal pain from an orthopedic and proctologic viewpoint; with a review of 180 cases. Am J Surg. (1950) 79(1):117–28. 10.1016/0002-9610(50)90202-9 15399361

[16] Postacchini F Massobrio M. Idiopathic coccygodynia. Analysis of fifty-one operative cases and a radiographic study of the normal coccyx. J Bone Joint Surg Am. (1983) 65(8):1116–24. doi: 10.2106/00004623-198365080-00011 6226668

[17] Nathan ST Fisher BE Roberts CS. Coccydynia: a review of pathoanatomy, aetiology, treatment and outcome. J Bone Joint Surg Br. (2010) 92(12):1622–27. 10.1302/0301-620X.92B12.25486 21119164

[18] Woon JTK Maigne J-Y Perumal V Stringer MD. Magnetic resonance imaging morphology and morphometry of the coccyx in coccydynia. Spine (Phila Pa 1976). (2013) 38(23):E1437–45. 10.1097/BRS.0b013e3182a45e07 23917643

[19] Manikandan R Iyer RD Iyer PR Shetty T AP Sri Vijayanand KS Kanna RM Predictors of outcomes of conservative management in chronic coccydynia - results from a prospective clinicoradiological observational study. J Orthop. (2024) 65:132–37. 10.1016/j.jor.2024.12.018 39906732 PMC11788802

[20] Foye PM. Coccydynia: tailbone pain. Phys Med Rehabil Clin N Am. (2017) 28(3):539–49. 10.1016/j.pmr.2017.03.006 28676363

[21] Sielatycki JA Metcalf T Chudik G Devin C Hodges SD Koscielski M. Understanding clinically relevant lumbar instability: a narrative review. J Am Osteopath Acad Orthop. (2022) VI(1). doi: 10.70709/azz81sdw4sh

[22] Garg B Ahuja K. Coccydynia-A comprehensive review on etiology, radiological features and management options. J Clin Orthop Trauma. (2020) 12(1):123–29. 10.1016/j.jcot.2020.09.025 33716437 PMC7920198

[23] Fogel GR Cunningham PY Esses SI. Coccygodynia: evaluation and management. J Am Acad Orthop Surg. (2004) 12(1):49–54. 10.5435/00124635-200401000-00007 14753797

[24] Maigne JY Lagauche D Doursounian L. Instability of the coccyx in coccydynia. J Bone Joint Surg Br. (2000) 82(7):1038–41. 10.1302/0301-620x.82b7.10596 11041598

[25] Moon SG Kim NR Choi JW Yi JG. Acute coccydynia related to precoccygeal calcific tendinitis. Skeletal Radiol. (2011) 41(4):473–76. 10.1007/s00256-011-1326-9 22109594

[26] Lirette LS Chaiban G Tolba R Eissa H. Coccydynia: an overview of the anatomy, etiology, and treatment of coccyx pain. Ochsner J. (2014) 14(1):84–7. 24688338 PMC3963058

[27] Berger O Lerner I Ohana G Sinelnikov I Talisman R. Desmoplastic neurotropic melanoma presenting as pilonidal Sinus: a rare clinical association. Am J Case Rep. (2021) 22:e932922. 10.12659/AJCR.932922 34429392 PMC8404162

[28] Lim Y Selbi W. Tarlov cyst. In: StatPearls. Treasure Island, FL: StatPearls Publishing (2023). Available online at: https://www.ncbi.nlm.nih.gov/books/NBK582154/ (Accessed June 12, 2023). 35881759

[29] Lee S-H Yang M Won H-S Kim Y-D. Coccydynia: anatomic origin and considerations regarding the effectiveness of injections for pain management. Korean J Pain. (2023) 36(3):272–80. 10.3344/kjp.23175 37394271 PMC10322656

[30] De Andrés J Chaves S. Coccygodynia: a proposal for an algorithm for treatment. J Pain. (2003) 4(5):257–66. 10.1016/s1526-5900(03)00620-5 14622695

[31] Won H-S Yang M Kim Y-D. Facet joint injections for management of low back pain: a clinically focused review. Anesth Pain Med (Seoul). (2020) 15(1):8–18. 10.17085/apm.2020.15.1.8 33329784 PMC7713865

[32] Wang S Hu L Yang X-L Yan T-T Xie C-L Wang S Evaluated anatomical variations in children by sacrococcygeal ultrasonography as a new tool: a report from a Chinese tertiary hospital. Ann Med. (2025) 57(1):2528977. 10.1080/07853890.2025.2528977 40624908 PMC12239231

[33] Gupta V Agarwal N Baruah BP. Magnetic resonance measurements of sacrococcygeal and intercoccygeal angles in normal participants and those with idiopathic coccydynia. Indian J Orthop. (2018) 52(4):353–57. 10.4103/ortho.IJOrtho_407_16 30078891 PMC6055471

[34] Trollegaard AM Aarby NS Hellberg S. Coccygectomy: an effective treatment option for chronic coccydynia: retrospective results in 41 consecutive patients. J Bone Joint Surg Br. (2010) 92(2):242–45. 10.1302/0301-620X.92B2.23030 20130316

[35] Yılmaz GG Tanrıverdi M Önal G Yiğit AB Şahin S Çakır FB. Understanding kinesiophobia in pediatric bone tumors: investigating its presence and predictive factors. Eur J Pediatr. (2025) 184(3):195. 10.1007/s00431-025-06032-9 39939522 PMC11821754

[36] Bain HL Mabrouk A Foye P. Coccyx pain. In: StatPearls. Treasure Island, FL: StatPearls Publishing (2025). Available online at: https://www.ncbi.nlm.nih.gov/sites/books/NBK563139/ (Accessed August 9, 2025). 33085286

[37] Hodges SD Eck JC Humphreys SC. A treatment and outcomes analysis of patients with coccydynia. Spine J. (2004) 4(2):138–40. 10.1016/j.spinee.2003.07.011 15016390

[38] Origo D Tarantino AG Nonis A Vismara L. Osteopathic manipulative treatment in chronic coccydynia: a case series. J Bodyw Mov Ther. (2017) 22(2):261–65. 10.1016/j.jbmt.2017.06.010 29861217

[39] Scott KM Fisher LW Bernstein IH Bradley MH. The treatment of chronic coccydynia and postcoccygectomy pain with pelvic floor physical therapy. PM R. (2016) 9(4):367–76. 10.1016/j.pmrj.2016.08.007 27565640

[40] Seker A Sarikaya IA Korkmaz O Yalcin S Malkoc M Bulbul AM. Management of persistent coccydynia with transrectal manipulation: results of a combined procedure. Eur Spine J. (2017) 27(5):1166–71. 10.1007/s00586-017-5399-6 29234884

[41] Blanco-Diaz M Palacios LR Martinez-Cerón MDR Perez-Dominguez B Diaz-Mohedo E. Physiotherapy approaches for coccydynia: evaluating effectiveness and clinical outcomes. BMC Musculoskelet Disord. (2025) 26(1):514. 10.1186/s12891-025-08744-3 40420056 PMC12105126

[42] Maigne J-Y Chatellier G Faou ML Archambeau M. The treatment of chronic coccydynia with intrarectal manipulation: a randomized controlled study. Spine (Phila Pa 1976). (2006) 31(18):E621–27. 10.1097/01.brs.0000231895.72380.64 16915077

[43] Nourani B Norton D Kuchera W Rabago D. Transrectal osteopathic manipulation treatment for chronic coccydynia: feasibility, acceptability and patient-oriented outcomes in a quality improvement project. J Osteopath Med. (2023) 124(2):77–83. 10.1515/jom-2023-0001 37999720

[44] Gönen Aydın C Örsçelik A Gök MC Akman YE. The efficacy of extracorporeal shock wave therapy for chronic coccydynia. Med Princ Pract. (2020) 29(5):444–50. 10.1159/000505835 31918431 PMC7511685

[45] Finsen V. [Corticosteroid injection for coccygodynia]. Tidsskr Nor Laegeforen. (2001) 121(24):2832–33. 11706491

[46] Gazioğlu Türkyılmaz G Rumeli Ş. Evaluation of the effectiveness of caudal epidural steroid injection a s an adjuvant to ganglion impar pulsed radiofrequency therapy in chron ic coccygodynia. Heliyon. (2024) 10(10):e31161. 10.1016/j.heliyon.2024.e31161 38778976 PMC11109874

[47] Kalstad AM Knobloch RG Finsen V. The treatment of coccydynia in adolescents: a case-control study. Bone Jt Open. (2020) 1(5):115–20. 10.1302/2633-1462.15.BJO-2020-0017 33225284 PMC7677095

[48] Sargin M Sari M Cicekci F Kara I. Retrospective evaluation of patients underwent ganglion Impar pulsed radiofrequency due to coccydynia. Sisli Etfal Hastan Tip Bul. (2022) 56(3):386–90. 10.14744/SEMB.2021.14396 36304226 PMC9580974

[49] Sencan S Edipoglu IS Ulku Demir FG Yolcu G Gunduz OH. Are steroids required in the treatment of ganglion impar blockade in chronic coccydynia? A prospective double-blinded clinical trial. Korean J Pain. (2019) 32(4):301–6. 10.3344/kjp.2019.32.4.301 31569923 PMC6813902

[50] Karaman H Tüfek A Kavak GO Yildirim ZB Celik F. Would pulsed radiofrequency applied to different anatomical regions ha ve effective results for chronic pain treatment? J Pak Med Assoc. (2011) 61(9):879–85. 22360028

[51] Sencan S Yolcu G Bilim S Kenis-Coskun O Gunduz OH. Comparison of treatment outcomes in chronic coccygodynia patients trea ted with ganglion impar blockade versus caudal epidural steroid injection: a prospective randomized comparison study. Korean J Pain. (2022) 35(1):106–13. 10.3344/kjp.2022.35.1.106 34966017 PMC8728552

[52] Ellinas H Sethna NF. Ganglion impar block for management of chronic coccydynia in an adolescent. Paediatr Anaesth. (2009) 19(11):1137–38. 10.1111/j.1460-9592.2009.03155.x 19807895

[53] Usmani H Dureja GP Andleeb R Tauheed N Asif N. Conventional radiofrequency thermocoagulation vs pulsed radiofrequency neuromodulation of ganglion Impar in chronic perineal pain of nononco logical origin. Pain Med. (2018) 19(12):2348–56. 10.1093/pm/pnx244 29329442

[54] Restrepo-Garces CE Saldarriaga NE Jaramillo S Gomez CM Vargas JF Ramirez LJ. Ganglion impar phenol injection in a pediatric patient with refractory cancer pain. Pain Med. (2014) 15(2):334–6. 10.1111/pme.12274 24224948

[55] Kuek DKC Chung SL Zishan US Papanikitas J Yanny S Meagher T Conus infarction after non-guided transcoccygeal ganglion impar block using particulate steroid for chronic coccydynia. Spinal Cord Ser Cases. (2019) 5:92. 10.1038/s41394-019-0237-1 31700690 PMC6831570

[56] Klocke R Jenkinson T Glew D. Sonographically guided caudal epidural steroid injections. J Ultrasound Med. (2003) 22(11):1229–32. 10.7863/jum.2003.22.11.1229 14620894

[57] Kao S-C Lin C-S. Caudal epidural block: an updated review of anatomy and techniques. Biomed Res Int. (2017) 2017:9217145. 10.1155/2017/9217145 28337460 PMC5346404

[58] Watanabe F Miyazu M Kojima T. Introduction of convenient conus to coccyx point-of-care ultrasound (C3PO) in children with a sacral dimple. J Clin Anesth. (2021) 74:110446. 10.1016/j.jclinane.2021.110446 34225186

[59] Kulkarni AG Tapashetti S Tambwekar VS. Outcomes of coccygectomy using the “Z” plasty technique of wound closure. Global Spine J. (2019) 9(8):802–6. 10.1177/2192568219831963 31819844 PMC6882096

[60] Kalstad AM Knobloch RG Finsen V. Coccygectomy in the treatment of chronic coccydynia. Spine (Phila Pa 1976). (2022) 47(10):E442–47. 10.1097/BRS.0000000000004209 34468434

[61] Perna A Franchini A Macchiarola L Maruccia F Barletta F Bosco F Coccygectomy for refractory coccydynia, old-fashioned but effective procedure: a retrospective analysis. Int Orthop. (2024) 48(8):2251–58. 10.1007/s00264-024-06236-y 38890180 PMC11246298

[62] Kara D Pulatkan A Ucan V Orujov S Elmadag M. Traumatic coccydynia patients benefit from coccygectomy more than patients undergoing coccygectomy for non-traumatic causes. J Orthop Surg Res. (2023) 18(1):802. 10.1186/s13018-023-04098-5 37891674 PMC10605957

[63] Hanley EN Ode G Jackson Iii BJ Seymour R. Coccygectomy for patients with chronic coccydynia: a prospective, observational study of 98 patients. Bone Joint J. (2016) 98-B(4):526–33. 10.1302/0301-620X.98B4.36641 27037436

[64] Frolov D Zhu K Rusev M Geiger K Fuller C Schmitz MA. Mind the gap: paramedian approach for coccygectomy. Spine J. (2024) 24(8):1424–30. 10.1016/j.spinee.2024.04.011 38643949

[65] Almetaher HA Mansour MA Shehata MA. Coccygectomy for chronic refractory coccygodynia in pediatric and adolescent patients. J Indian Assoc Pediatr Surg. (2021) 26(2):102–6. 10.4103/jiaps.JIAPS_22_20 34083893 PMC8152407

[66] Finsen V Kalstad A Knobloch RG. Normal preoperative images do not indicate a poor outcome of surgery for coccydynia. Spine (Phila Pa 1976). (2020) 45(22):1567–71. 10.1097/BRS.0000000000003642 33122606

[67] Milosevic S Andersen GØ Jensen MM Rasmussen MM Carreon L Andersen MØ The efficacy of coccygectomy in patients with persistent coccydynia. Bone Joint J. (2021) 103-B(3):542–46. 10.1302/0301-620X.103B3.BJJ-2020-1045.R2 33641429

[68] Roa JA White S Barthélemy EJ Jenkins A Margetis K. Minimally invasive endoscopic approach to perform complete coccygectom y in patients with chronic refractory coccydynia: illustrative case. J Neurosurg Case Lessons. (2022) 3(3):CASE21533. 10.3171/CASE21533 36130572 PMC9379649

[69] Dean LM Syed MI Jan SA Patel NA Shaikh A Morar K Coccygeoplasty: treatment for fractures of the coccyx. J Vasc Interv Radiol. (2006) 17(5):909–12. 10.1097/01.RVI.0000217953.74013.87 16687760

[70] Akar E Koban O Öğrenci A Yılmaz M Dalbayrak S. Polymethylmetacrylate cement augmentation of the coccyx (coccygeoplast y) for fracture: a case report. Balkan Med J. (2020) 37(6):348–50. 10.4274/balkanmedj.galenos.2020.2020.4.68 32573177 PMC7590546

[71] Manfre L Gil I Baptista T Calvão Pires P de Vivo AE Masala S Coccygeoplasty: preliminary experience with this new alternative treatment of refractory coccydynia in patients with coccyx hypermobility. J Neurointerv Surg. (2022) 15(1):82–5. 10.1136/neurintsurg-2021-018149 35882554

